# Physician readiness for artificial intelligence integration in clinical screening and diagnosis: Multicenter cross-sectional study in Jeddah, Saudi Arabia

**DOI:** 10.1097/MD.0000000000050871

**Published:** 2026-09-25

**Authors:** Haneen Abu Alsoud, Abdulhameed Abu Alsoud, Abdulelah K. Alqawlaq, Abdulrahim Alissa, Malek Odah, Mohamed Abouassad, Lin Mazen Jolha, Rebal Najeeb Khayyat, Rian Saeed Yahya, Renad Ashraf Altaher, Lujain Waleed Alqabbaa, Joud Abdullah Albassam, Ehab A. Abo-Ali, Fatma E. Hassan

**Affiliations:** a General Medicine Practice Program, Batterjee Medical College, Jeddah, Saudi Arabia; b Emergency Medicine Department, Rashid Hospital, Dubai, United Arab Emirates; c Department of Community Medicine, General Medicine Practice Program, Batterjee Medical College, Jeddah, Saudi Arabia; d Public Health and Community Medicine Department, Faculty of Medicine, University of Tanta, Tanta, Egypt; e Department of Physiology, General Medicine Practice Program, Batterjee Medical College, Jeddah, Saudi Arabia; f Medical Physiology Department, Faculty of Medicine, Kasr Alainy, Cairo University, Giza, Egypt.

**Keywords:** artificial intelligence, attitude, barriers, clinical practice, knowledge, physicians, Saudi Arabia

## Abstract

Artificial intelligence (AI) is increasingly incorporated into clinical screening and diagnostic workflows worldwide. However, the effectiveness and safety of AI implementation depend largely on physicians’ readiness, including their knowledge, attitudes, and perceived barriers. In Saudi Arabia, where healthcare digital transformation is a strategic priority under Vision 2030, evidence regarding physician preparedness for AI-enabled clinical practice remains limited. A multicenter cross-sectional study was conducted among physicians and medical interns working in governmental and private hospitals in Jeddah, Saudi Arabia, between February and April 2025. Data were collected using a validated, self-administered questionnaire assessing demographic characteristics, knowledge, attitudes, and perceived barriers related to AI use in clinical screening and diagnosis. Descriptive statistics, chi-square tests, and multivariate logistic regression analyses were performed using International Business Machines Statistical Package for the Social Sciences version 26.0. A total of 435 physicians participated in the study. Although most respondents reported positive attitudes toward AI integration in clinical practice (83.7%), only half demonstrated good knowledge of AI concepts and applications (50.3%), indicating a clear readiness gap. Radiology (75.9%) and pathology (43.7%) were identified as the clinical areas with the greatest perceived potential for AI implementation. The most frequently reported barriers were insufficient training (58.9%) and lack of trust in AI systems (53.8%). In multivariate analysis, non-Saudi physicians were significantly more likely to exhibit positive attitudes toward AI adoption (adjusted odds ratio = 1.75, *P* = .047). Despite strong physician enthusiasm for AI, substantial gaps in knowledge and training persist, posing challenges to effective implementation in clinical practice. Addressing physician readiness through structured educational programs, trust-building strategies, and clear regulatory frameworks is essential to ensure safe, sustainable, and effective integration of AI into Saudi healthcare systems.

## 1. Introduction

Integrating artificial intelligence (AI) into healthcare is transforming clinical screening and diagnostic processes worldwide, offering opportunities for improved accuracy and efficiency. Advanced AI methodologies, including machine learning and deep learning, are increasingly applied in image-based specialties such as radiology, pathology, and ophthalmology, where they have demonstrated diagnostic performance comparable to, and in some cases exceeding, that of human clinicians.^[1–4]^

In Saudi Arabia, AI is increasingly recognized as a key driver of healthcare reform under the Kingdom’s Vision 2030 initiative, which prioritizes digital transformation. Several practical applications of AI are already being explored in the country. For example, institutions such as King Abdulaziz University Hospital in Jeddah are investigating AI tools in pathology to assist in histopathological cancer diagnoses, which could enhance diagnostic precision and treatment planning.^[5,6]^ Additionally, AI-powered telemedicine solutions are beginning to play a critical role in expanding healthcare access in underserved regions, demonstrating AI’s ability to address geographic disparities in care delivery.^[4]^ While these applications showcase the potential of AI, they also highlight the importance of addressing adoption barriers to fully realize its benefits.^[2,5]^

Despite these advancements, significant challenges continue to limit the widespread adoption of AI in clinical practice. Ethical concerns, such as accountability in AI decision-making, data privacy, and the potential for algorithmic bias, have raised questions about trust and transparency in AI systems.^[1,2,6]^ In Saudi Arabia, these challenges are compounded by gaps in regulatory frameworks and disparities in infrastructure and expertise across healthcare facilities.^[2,5]^ For example, while AI has demonstrated efficacy in reducing diagnostic errors, concerns about patients’ data security and the readiness of healthcare professionals to adopt AI remain significant obstacles to its implementation. Addressing these barriers will require a combination of clear ethical guidelines, potent regulatory systems, and comprehensive training programs tailored to the needs of Saudi healthcare professionals.

### 1.1. Literature review

AI has increasingly been incorporated into healthcare to support screening, diagnosis, clinical decision-making, and workflow optimization. Machine-learning and deep-learning approaches have demonstrated particular value in image-intensive specialties such as radiology, pathology, and ophthalmology, where AI systems can assist clinicians in identifying abnormalities and interpreting complex diagnostic data.^[1–4]^ However, successful clinical implementation depends not only on technological performance but also on clinicians’ knowledge, acceptance, trust, and ability to use AI appropriately.

Within Saudi Arabia, previous studies have generally reported favorable attitudes toward AI while simultaneously identifying gaps in knowledge, practical exposure, and training. Serbaya et al assessed healthcare workers in private polyclinics in Jeddah and found generally good awareness and optimistic attitudes, but limited AI-related behaviors in practice.^[5]^ Among physicians in 2 major hospitals in Jeddah, Alkhatieb and Subke similarly reported favorable perceptions of clinical AI, although only a minority had used AI systems during the preceding year.^[7]^ Alabbad et al also reported high AI awareness among medical students, interns, and residents in Riyadh, while identifying important gaps in knowledge of AI subtypes and medical applications.^[8]^ Together, these findings suggest that enthusiasm for AI does not necessarily indicate practical readiness for routine clinical implementation.

Previous Saudi research has also identified barriers that may influence readiness for AI adoption. Insufficient education and training, limited hands-on exposure, concerns regarding reliability and transparency, data privacy, and uncertainty regarding regulation and accountability have been repeatedly described.^[6,9]^ Educational exposure appears particularly relevant; Alwadaani reported greater AI knowledge among medical students and doctors who had received AI-related education than among those without such exposure.^[8]^ These findings support the need to examine physician knowledge and attitudes together with the practical, educational, ethical, and organizational factors that may influence AI adoption.

To clarify how the present study extends the existing evidence, Table 1 summarizes representative Saudi Arabian studies addressing healthcare professionals’ knowledge, attitudes, perceptions, readiness, and concerns regarding AI. The studies differ substantially in population, setting, and outcomes assessed, allowing the remaining research gap to be identified more explicitly.

**Table 1:** State of the art and research gap in Saudi Arabian studies of healthcare professionals’ readiness for AI.

Study	Setting and population	Main focus and contribution	Research gap relative to the present study
Elnaggar et al, 2023^[9]^	1026 healthcare providers across Saudi health facilities.	Assessed perceptions and worries regarding AI and reported generally favorable perceptions with moderate knowledge of AI applications.	Included a heterogeneous healthcare workforce and evaluated AI broadly rather than physician readiness specifically for AI-assisted clinical screening and diagnosis.
Serbaya et al, 2024^[5]^	361 healthcare workers in private polyclinics in Jeddah; physicians represented approximately one-quarter of participants.	Evaluated awareness, attitudes, and practices; identified generally good awareness and optimistic attitudes but limited AI-related behavior in practice.	Restricted to private polyclinics and a mixed healthcare workforce; did not provide a physician-focused assessment across governmental and private hospital settings.
Alkhatieb and Subke, 2024^[7]^	205 physicians from 2 major hospitals in Jeddah.	Examined physicians’ attitudes, perceptions, and utilization of clinical AI; favorable perceptions contrasted with low recent AI use.	Focused principally on attitudes, perceptions, and use; did not comprehensively combine knowledge, barriers, training preferences, and accountability for AI-related diagnostic errors.
Alabbad et al, 2025^[8]^	159 medical students and doctors at King Faisal University, Al-Ahsa.	Assessed knowledge, perceptions, practice, and educational exposure; AI education was associated with greater knowledge.	Single-university population combining students and doctors; not specifically focused on physician readiness for AI-assisted screening and diagnosis.
Alsaedi et al, 2024^[6]^	314 licensed healthcare professionals across Saudi health facilities.	Examined AI awareness, previous experience, and adoption-related concerns, including data management and regulatory/ethical worries.	Included multiple healthcare professions and concentrated primarily on concerns surrounding AI adoption rather than a comprehensive physician readiness assessment.
Alabbad et al, 2025^[8]^	374 medical students, interns, and residents in Riyadh.	Evaluated AI knowledge, attitudes, and practice; showed high awareness and favorable attitudes alongside important knowledge and educational gaps.	Population centered on students and physicians in training; did not comprehensively examine physicians across career levels together with barriers, training preferences, and accountability.
Alnajdi et al, 2026^[10]^	Systematic review of 22 Saudi studies published from 2023 to 2026.	Synthesized healthcare professionals’ readiness, perceptions, and barriers; found generally positive perceptions but only moderate practical readiness, limited hands-on experience, and low formal training.	Synthesized heterogeneous healthcare professions and AI applications; did not provide new physician-level primary data specifically focused on readiness for clinical screening and diagnostic integration.
Present study	435 physicians and medical interns from governmental and private hospitals in Jeddah.	Integrates knowledge, attitudes, perceived clinical applications, barriers, training preferences, accountability, and multivariable analysis of factors associated with readiness.	Addresses the need for a multicenter, physician-focused assessment integrating multiple implementation dimensions directly relevant to AI-assisted clinical screening and diagnosis.

AI = artificial intelligence.

As shown in Table 1, Saudi Arabian research has consistently identified favorable perceptions of AI alongside limitations in knowledge, training, practical exposure, or confidence in implementation. A recent systematic review of 22 Saudi studies similarly concluded that healthcare professionals generally view AI positively, while practical readiness remains moderate and formal training and hands-on experience are limited.^[10]^ Nevertheless, evidence specifically integrating physicians’ knowledge and attitudes with perceived implementation barriers, preferred training strategies, perceived clinical areas for AI application, and accountability for AI-related diagnostic errors remains limited. The present study addresses this gap through a multicenter assessment of physicians and medical interns from governmental and private hospitals, with a specific focus on readiness for AI integration into clinical screening and diagnostic practice.

Therefore, this multicenter study aims to assess physician readiness for AI integration by evaluating knowledge levels, attitudes, perceived clinical applications, barriers to adoption, preferred training strategies, and perceptions of accountability among physicians and medical interns working in Jeddah, Saudi Arabia. By identifying critical gaps in preparedness, this study provides actionable insights to inform workforce training, regulatory planning, and implementation strategies needed to support the safe and effective adoption of AI in clinical practice.

## 2. Materials and methods

### 2.1. Ethical approval

This study was approved by the ethics committee at Batterjee Medical College in Jeddah, Saudi Arabia (RES-2025-0002). Following international ethical guidelines, participation was voluntary, and electronic written informed consent was obtained from all participants before they accessed the questionnaire. Participants first viewed an information page describing the study and indicated their agreement by selecting an “I agree to participate” option. This consent procedure, including the use of electronic consent, was reviewed and approved by the Batterjee Medical College Ethics Committee (RES-2025-0002).

### 2.2. Study design and setting

We conducted a cross-sectional, multicenter study between February 20, 2025, and April 22, 2025, among physicians and interns from various governmental and private hospitals in Jeddah, Saudi Arabia.

### 2.3. Questionnaire tool

Data were collected using a self-administered, closed-ended online survey disseminated through WhatsApp to the target population. The validity of the questionnaire was rigorously assessed through multiple methods. Initially, content validity was ensured by developing items based on a comprehensive literature review and expert consultations. Construct validity was evaluated using exploratory factor analysis, which confirmed the expected factor structure, with items loading on their respective constructs. Cronbach alpha test result was 0.84. A pilot study with 30 participants was conducted to refine the instrument, ensuring the clarity and relevance of the items and the reliability of the questionnaire. All statistical analyses were performed using International Business Machines Statistical Package for the Social Sciences version 26.0 (IBM Corp., Armonk), which included confirmatory factor analysis and correlation analysis. The questionnaire was in the English language and consisted of 6 sections: consent, demographics, knowledge of AI, attitudes toward AI, ethical concerns, and future perspectives (See Supplemental Digital Content 1).

### 2.4. Study population (inclusion and exclusion criteria)

Eligible participants in this study included physicians and medical interns residing in Jeddah, age above 25 years either male or female who consented to participate. Medical students were excluded from the study due to their limited experience in using AI in clinical settings.

### 2.5. Sample size and data collection

The smallest range of samples was determined to be 384, based on a 95% confidence level, an estimated incidence of 50%, and a 5% allowable error margin. The sample size formula used was S = Z^2^ × P × (1 − P)/ M^2^. [S: sample size, Z: Z-score corresponding to 95% confidence level, P: estimated population proportion (0.5), and M: margin of error (0.05)].^[11]^ A total of 435 responses were collected between February 20, 2025, and April 22, 2025.

### 2.6. Statistical analysis

The Statistical Package for the Social Sciences 26 software was used for data analysis. Frequencies and percentages summarized the categorical variables, while means and standard deviations expressed continuous variables, and a *t*-test was used. In contrast, associations between categorical variables were assessed using the chi-square test (or Fisher exact test when appropriate). A *P* value ≤ .05 was considered statistically significant. All data were stored securely on password-protected devices accessible only to the research team. After the study was completed, the data were backed up on encrypted external storage to be retained for 5 years in accordance with institutional data management policies before being securely deleted.

## 3. Results

As described in Table 2, the number of participating healthcare workers was 435. Most were females (52.4%). The majority aged 25 to 34 years (56.3%). The prevalence of non-Saudi participants was slightly higher than that of Saudi participants (53.6% vs 46.4%). Approximately half of the respondents (47.4%) had clinical experience of < 5 years. Based on job titles, medical interns formed the majority (32.6%), followed by general practitioners (22.1%) and specialists (21.1%).

**Table 2:** Descriptive analysis of demographics.

Variables (n = 435)	Frequency	Percentage (%)
Gender		
Male	207	47.6
Female	228	52.4
Age group		
25–34	245	56.3
> 34–44	113	26.0
> 44	77	17.7
Nationality		
Saudi	202	46.4
Non-Saudi	233	53.6
Job title		
Medical intern	142	32.6
General practitioner	96	22.1
Resident	38	8.7
Specialist	92	21.1
Consultant	67	15.4
Years of experience in the medical field		
< 5 yrs	206	47.4
5–10 yrs	90	20.7
> 10 yrs	139	32.0

n = number of individuals.

Among the participants, 219 (50.3%) showed good knowledge of AI in medical screening and diagnosis, while 216 (49.7%) showed poor knowledge. Regarding attitude, the overwhelming majority (83.7%) had a positive attitude towards integrating AIinto clinical practice, compared to 71 (16.3%) who had a negative attitude, as seen in Figure 1.

**Figure 1.**
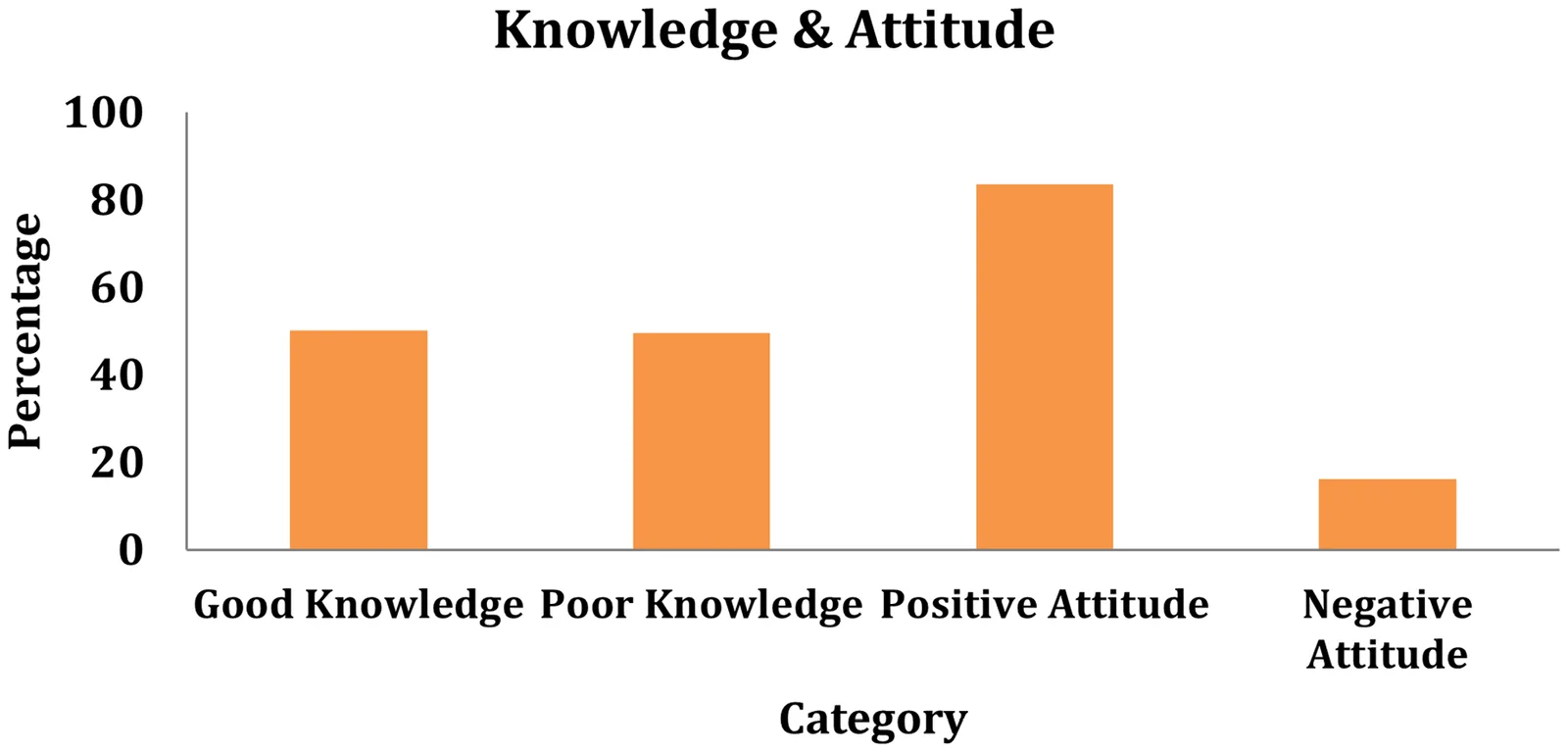
Distribution of knowledge and attitude levels regarding AI in screening and diagnosis. AI = artificial intelligence.

Using chi-square analysis, the knowledge levels were significantly related to years of work experience (*P* = .049), and participants with < 5 years of experience were more likely to demonstrate good knowledge. Other factors, including gender, age category, nationality, and occupation, were reported to have no significant association with knowledge levels.

On the other hand, gender (*P* = .043) and nationality (*P* = .019) were strongly associated with attitude. Non-Saudi physicians and males were significantly more likely to have a positive attitude towards integrating AI in screening and diagnosis. No statistically significant relationships were found between attitude, age, position, and years of experience, as revealed in Table 3.

**Table 3:** Sociodemographic factors relationship with knowledge & attitude.

Variables sociodemographics & socio-occupational	Total (n = 435)	Knowledge		*P* value	Attitude		*P* value
		Poor (n)	Good (n)		Negative (n)	Positive (n)	
Gender							
Male	207	98	109	.358	26	181	.043*
Female	228	118	110		45	183	
Nationality							
Saudi	202	97	105	.525	42	160	.019*
Non-Saudi	233	119	114		29	204	
Age group							
25–34	245	112	133	.114	46	199	.290
> 34–44	113	65	48		15	98	
> 44	77	39	38		10	67	
Job title							
Medical intern	142	64	78	.439	31	111	.215
General physician	96	48	48		16	80	
Resident	38	17	21		4	34	
Specialist	92	48	44		11	81	
Consultant	67	39	28		9	58	
Years of experience							
< 5	206	90	116	.049*	34	172	.263
5–10	90	52	38		19	71	
> 10	139	74	65		18	121	

n = number of individuals.

* : Statistically significant.

Multivariate logistic regression analysis was performed for all demographic variables in relation to knowledge and attitude levels. As shown in Table 4, none of the demographic factors were identified as significant predictors of knowledge (*P* > .05). On the other hand, nationality was found to be a significant predictor of positive attitude (*P* = .047; adjusted odds ratio = 1.748; 95% confidence interval: 1.007–3.036), with non-Saudi physicians being more likely to have a positive attitude toward AI in clinical practice.

**Table 4:** Regression analysis of socio-demographics variables with knowledge and attitude.

Variable	Knowledge			Attitude		
	aOR	95% CI	*P* value	aOR	95% CI	*P* value
Gender						
Male (ref)	1			1		
Female	0.771	0.514–1.155	.207	0.678	0.389–1.181	.170
Nationality						
Saudi (ref)	1			1		
Non-Saudi	0.893	0.593–1.345	.589	1.748	1.007–3.036	.047*
Age group						
25–34 (ref)	1			1		
35–44	0.782	0.426–1.435	.427	1.340	0.586–3.064	.489
> 45	1.104	0.470–2.591	.821	1.148	0.345–3.822	.822
Job title						
Medical intern (ref)	1			1		
General practitioner	0.991	0.564–1.740	.975	1.402	0.665–2.955	.374
Resident	1.120	0.526–2.387	.769	2.927	0.913–9.390	.071
Specialist	0.954	0.477–1.906	.894	2.135	0.825–5.526	.118
Consultant	0.641	0.272–1.512	.310	1.984	0.596–6.600	.264
Years of experience						
5 years (ref)	1			1		
5–10 years	0.614	0.350–1.076	.089	0.502	0.244–1.033	.061
> 10 years	0.820	0.381–1.763	.611	0.565	0.194–1.645	.295

aOR = adjusted odds ratio, CI = confidence interval, ref = reference.

* : Statistically significant.

In addition to knowledge and attitudes, participants shared their perspectives on AI’s diagnostic potential, barriers to adoption, best strategies for training, and accountability for AI-related errors. As shown in Figure 2, most respondents perceived the greatest potential for AI implementation in radiology (75.9%), followed by pathology (43.7%) and primary care (31.5%), while lower perceived potential was reported for oncology (28.7%), surgery (25.5%), cardiology (23.9%), neurology (22.8%), and mental health (18.6%). Figure 3 illustrates the main barriers to AI adoption in clinical practice, with insufficient training or education (58.9%) and lack of trust in AI systems (53.8%) being the most frequently reported challenges, followed by cost (48.7%), lack of clear evidence of efficacy (46.4%), and data privacy concerns (43.7%), whereas legal barriers and resistance from leadership were less commonly reported. Preferred strategies for familiarizing healthcare professionals with AI tools are presented in Figure 4, where online courses or webinars (51.7%) and AI-focused conferences or seminars (50.8%) were most commonly endorsed, followed by integration of AI training into medical school or residency curricula (48.3%) and engagement through research and publications (43.0%). Finally, Figure 5 summarizes physicians’ perspectives regarding accountability for diagnostic errors involving AI-based tools, with most respondents assigning responsibility to healthcare providers (41.6%), followed by AI developers (29.0%) and healthcare institutions or hospitals (19.5%), while a minority believed that no party should be held accountable (9.9%).

**Figure 2.**
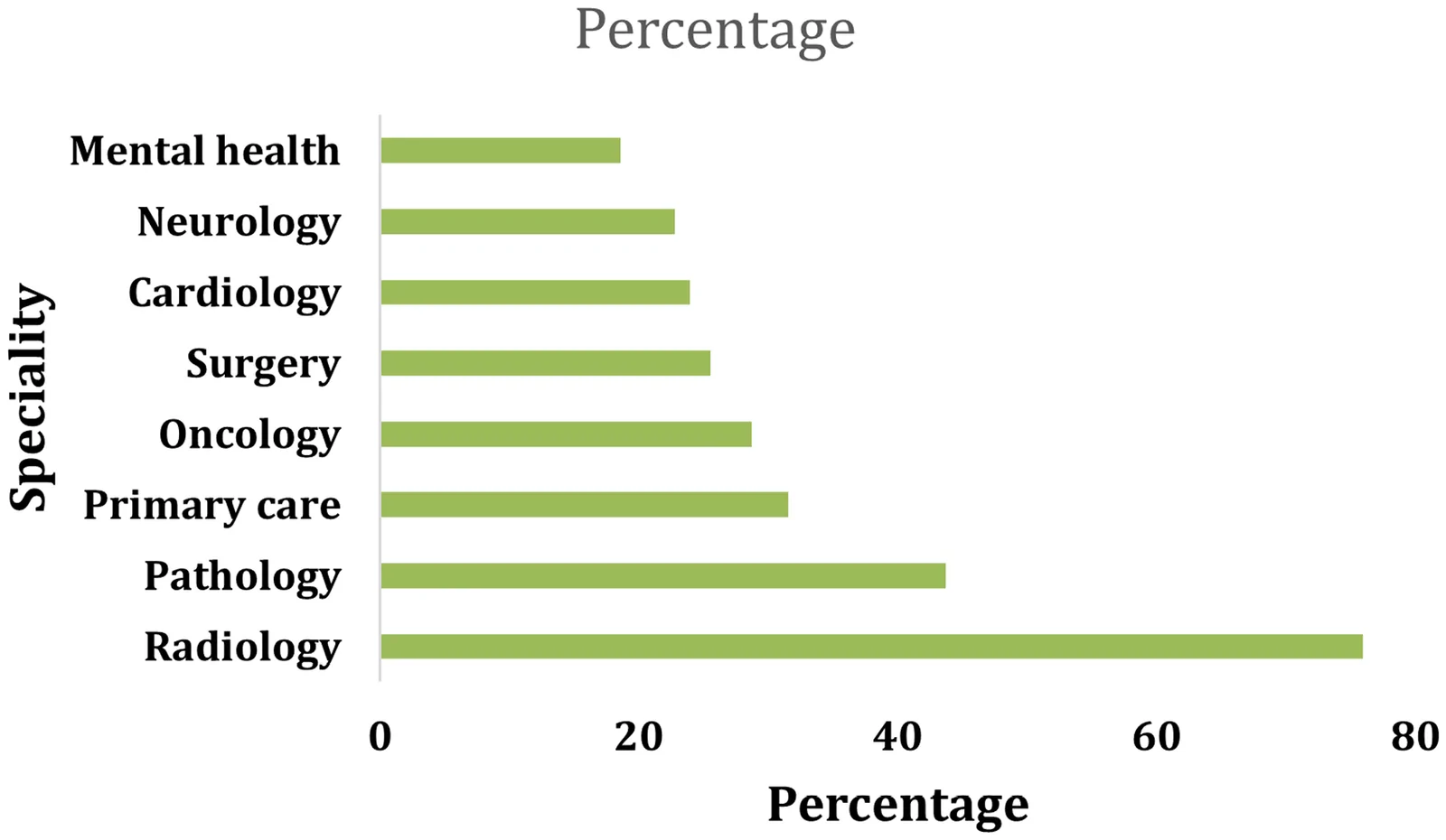
Perceived potential of AI across clinical diagnostic specialties. AI = artificial intelligence.

**Figure 3.**
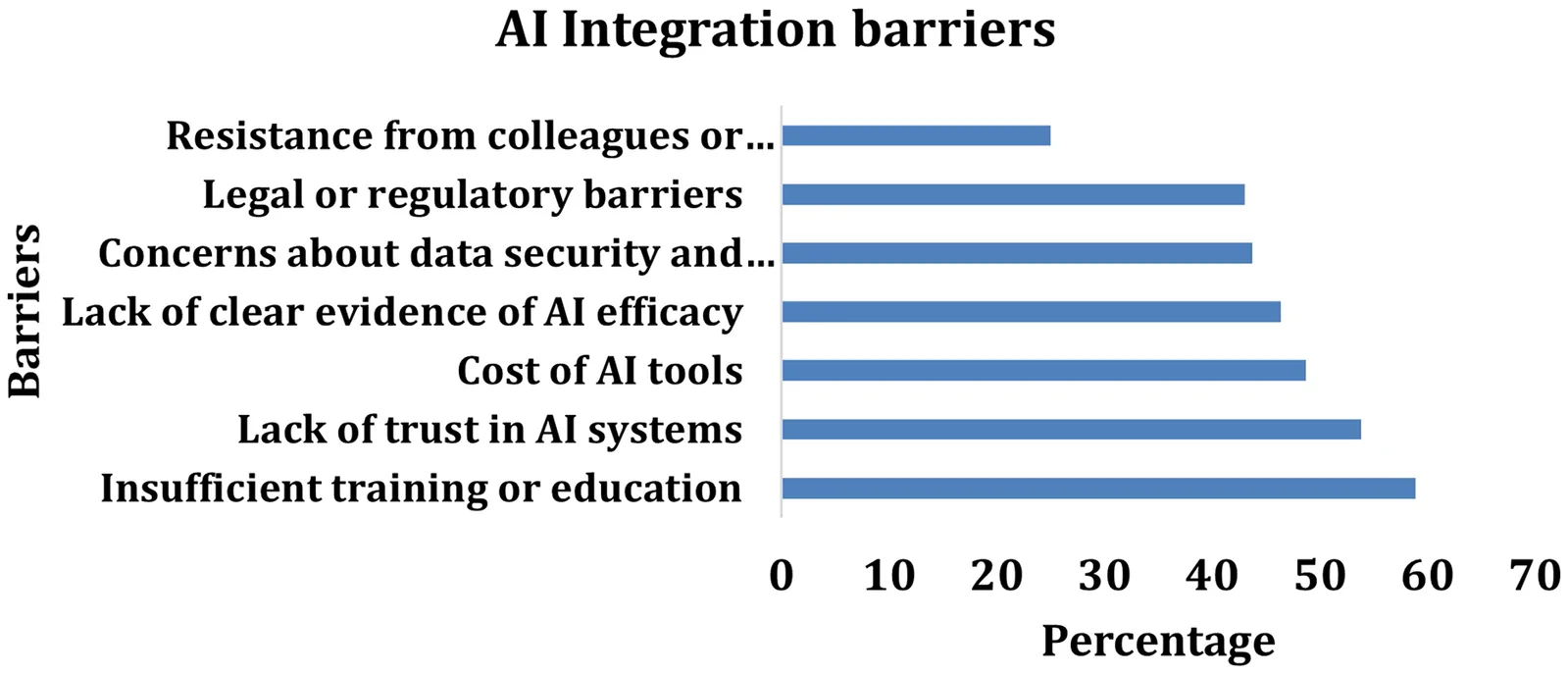
Reported barriers to the adoption of AI in clinical practice. AI = artificial intelligence.

**Figure 4.**
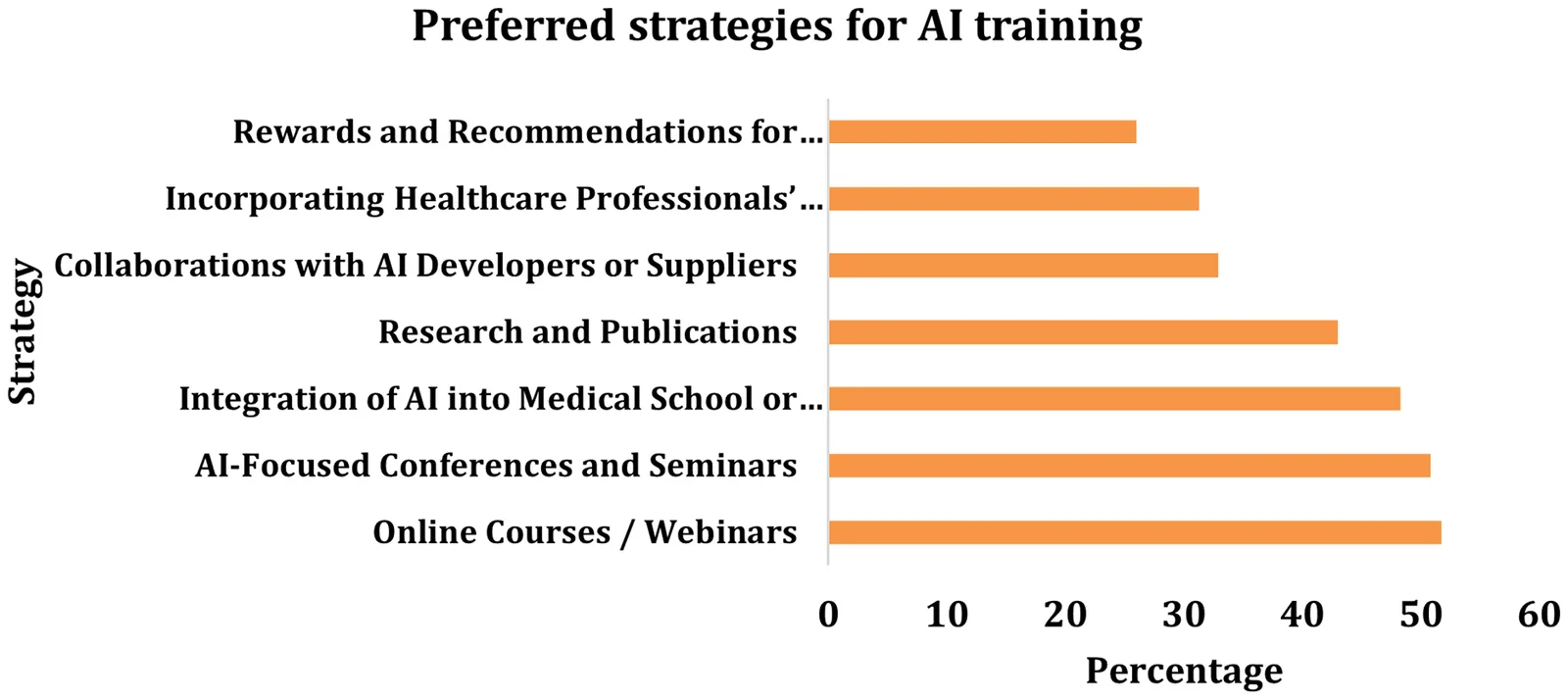
Preferred strategies for training and familiarizing healthcare professionals with AI tools. AI = artificial intelligence.

**Figure 5.**
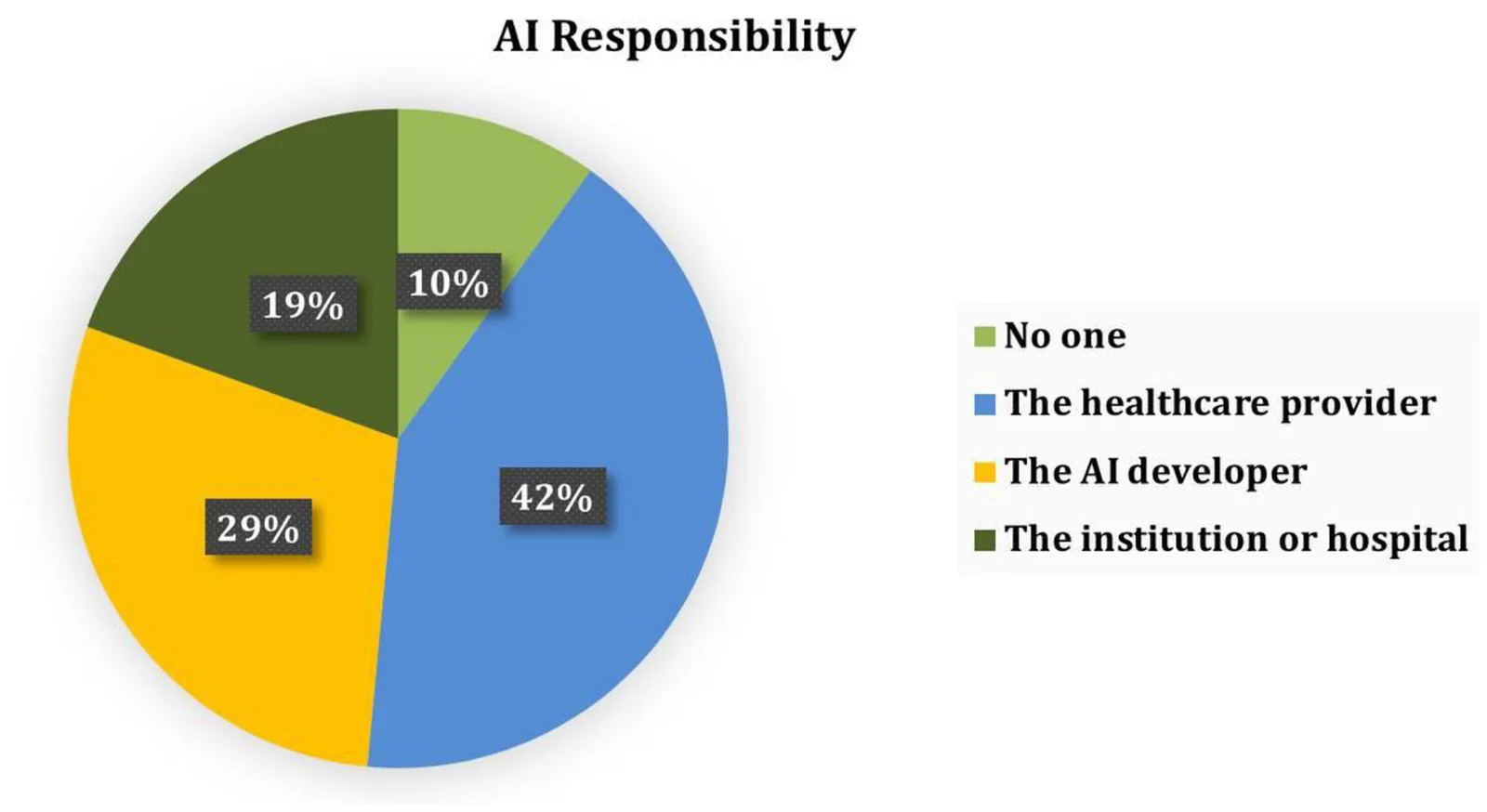
Physicians’ perspectives on accountability for diagnostic errors involving AI-based tools. AI = artificial intelligence.

## 4. Discussion

This multicenter study highlights a critical readiness gap among physicians in Jeddah, Saudi Arabia, regarding the integration of AI into clinical screening and diagnostic practice. While the majority of physicians expressed positive attitudes toward AI, only half demonstrated adequate knowledge of AI concepts and applications, revealing a clear disconnect between enthusiasm and preparedness. This gap represents a significant challenge to the safe and effective implementation of AI in routine clinical care, particularly in healthcare systems undergoing rapid digital transformation.

This study adds to the emerging body of literature on AI in healthcare by providing real-world, system-level insights into physician readiness, perceived barriers, and implementation needs within a rapidly digitizing healthcare system.

The coexistence of high optimism and limited knowledge observed in this study is consistent with findings reported in other regions of Saudi Arabia and internationally.^[1–3,7–9]^ Similar studies have shown that healthcare professionals often perceive AI as a valuable supportive tool, yet lack sufficient understanding of its operational principles, limitations, and ethical implications.^[1,2,6]^ Without targeted training and structured implementation strategies, this imbalance risks leading to underutilization, inappropriate reliance, or mistrust of AI technologies in clinical decision-making.^[4]^ This reflects a broader national trend where awareness of AI outpaces actual functional understanding. This gap highlights the need for targeted educational interventions. Without a deeper understanding of AI’s operational frameworks, even the strongest enthusiasm for the technology is unlikely to translate into widespread clinical adoption. This finding aligns with international studies highlighting a consistent gap between enthusiasm for AI and the reality of literacy in the field.^[5,6]^

Insufficient training and lack of trust emerged as the most prominent barriers to AI adoption in this study, reflecting a broader challenge in translating technological innovation into routine clinical practice. More than half of the participating physicians identified inadequate education and limited hands-on exposure as major obstacles, highlighting a gap between theoretical awareness of AI and practical readiness to use it safely in clinical decision-making. Similar barriers have been reported in previous studies conducted in Saudi Arabia and internationally, where healthcare professionals expressed enthusiasm toward AI but reported uncertainty regarding its reliability, interpretability, and ethical use in patient care.^[5–9]^

Trust in AI systems remains a critical determinant of adoption, particularly in high-stakes clinical environments such as screening and diagnosis. Concerns related to algorithm transparency, accountability for diagnostic errors, and data privacy continue to undermine physician confidence in AI-assisted tools.^[1,2,6]^ Without clear regulatory frameworks and defined medico-legal responsibilities, physicians may be reluctant to rely on AI outputs, even when such systems demonstrate high diagnostic accuracy. These findings align with existing literature emphasizing that trust in AI is shaped not only by technical performance but also by governance structures, ethical safeguards, and clinician involvement in implementation processes.^[6,7,9]^

Addressing these barriers requires a multifaceted approach centered on workforce development and system-level planning. Integrating structured AI education into undergraduate medical curricula, residency training, and continuing professional development programs is essential to improve physician competence and confidence.^[7,8]^ In parallel, national regulatory bodies should establish clear guidelines outlining standards for AI validation, accountability, and clinical oversight to support safe and responsible use. Within the context of Saudi Vision 2030, strengthening physician readiness through targeted training and supportive governance represents a critical step toward ensuring that AI technologies are implemented effectively, equitably, and sustainably across the healthcare system.

The positive attitude described in our study among Jeddah physicians was consistent with findings from other regions of Saudi Arabia. For example, a study at King Faisal University, Al-Ahsa, found that most participants, both those who received and those who did not receive AI education, agreed or strongly agreed that AI is an integral part of medical practice.^[8]^ Similarly, Alabbad et al found that more than 4 out of 5 medical interns and physicians in Riyadh considered AI useful in diagnosis.^[8]^ However, noticeable anxieties temper the excitement surrounding AI. In a study conducted among pilgrims in Makkah, 73.7% expressed uncertainty when it comes to fully trusting AI with their lives.^[12]^ This highlights a complex relationship with AI: while people are generally open to using AI as a tool to enhance efficiency, they are more skeptical about allowing AI to make decisions independently.

Demographics seem to be driving attitudes toward AI. Our finding that male physicians showed a more positive attitude towards AI is consistent with another study by Alkhatieb in Jeddah, who also found a statistically significant relationship between male physicians and a positive attitude (*P* = .01).^[7]^ Our results also identified that non-Saudi physicians expressed a more positive attitude towards AI. This may be linked to differing educational systems or greater prior exposure to AI-integrated clinical environments, highlighting a key area for future investigation. Comparable demographic trends in AI perception have been documented in multiple cross-country studies, suggesting that previous training and experience with systems have a significant impact on how receptive individuals are to AI.^[8]^

One of the key themes appearing from regional studies is the significant gap between attitude and actual use. Alkhatieb recently reported that a high proportion of Jeddah physicians (76%) trusted diagnoses made through AI; however, only 25.9% had interacted with AI systems in the last 12 months.^[7]^ Similar results were found in a study conducted by Alabbad et al in Riyadh, Saudi Arabia, which showed that respondents had a positive attitude towards AI; however, a total of 77.8% of the participants showed poor knowledge.^[8]^ Our results indicate that high optimism, coupled with moderate knowledge, helps explain this gap between attitude and practice. Enthusiasm alone is insufficient; a lack of concrete knowledge and training slows successful implementation.

To bridge this gap, educational interventions are essential. Alwadaani and his colleagues, in Al-Ahsa, documented that participants who received formal AI training had significantly more knowledge of its basic concepts, subtypes, and applications in medical practice (96.9% vs 81.1%). Additionally, trained professionals were much more likely to report using AI in their practice (75% vs 12.6%).^[8]^ Therefore, integrating comprehensive AI curricula into medical schools and residency programs is not just advisable but necessary. Improving physicians’ confidence and competence in AI will help Saudi Arabia to move from an interest stage to active incorporation, aligning with the Vision 2030 goals and enhancing healthcare delivery. Furthermore, evidence from systematic reviews supports the integration of AI training programs at undergraduate and postgraduate levels.^[7,13]^

Moreover, our findings provided important insights into how physicians in Jeddah, Saudi Arabia, Jeddah, view AI’s potential and its barriers. Most respondents believed AI would have the most significant impact in radiology and pathology (75.9% and 43.7%), reflecting global trends in which image-based specialties have pioneered AI adoption.^[14]^ In particular, deep learning has been shown to revolutionize radiology practices, providing performance comparable to human experts.^[15]^ However, significant barriers persist, particularly insufficient training (58.9%) and a lack of trust (53.8%), both of which mirror findings from previous national and international studies.^[16,17]^ Similar concerns were highlighted by Saudi healthcare providers in other research, where worries about AI adoption were prevalent.^[18]^ Respondents also emphasized the importance of formal training, online courses, conferences, and curricular integration to accelerate the adoption of AI into the medical field, similar to what has been highlighted in previous national and international research.^[19]^

Finally, many respondents (41.6%) believed healthcare providers should take responsibility if AI-based diagnostic tools make an incorrect diagnosis, while fewer attributed responsibility to AI developers or health institutions (29% and 19.5%). This raises ethical and legal issues that need to be resolved in order to create trust in AI-based clinical decisions.^[20–26]^ These findings emphasize the critical need for regulatory frameworks, continuous training, and clear guidelines to foster the adoption of AI.^[27–34]^

Our study also addressed the issue of sample size and study reliability. The determination of our sample size was grounded in established statistical guidance,^[11]^ which enhances the credibility of our conclusions despite being a cross-sectional design.

These findings have important implications for multiple stakeholders involved in the implementation of AI in healthcare. Policymakers may use this evidence to inform national AI governance frameworks and regulatory standards that support safe and accountable clinical use. Medical educators can leverage these insights to integrate AI competencies into undergraduate and postgraduate medical curricula, ensuring that future clinicians are adequately prepared for AI-assisted practice. Healthcare institutions may apply these findings to develop structured implementation and training strategies that enhance clinician trust and adoption, while AI developers can align system design with physicians’ expectations to improve usability, transparency, and clinical relevance.

### 4.1. Strengths, limitations, and future research

This study has several notable strengths. It represents one of the few multicenter investigations in Saudi Arabia specifically examining physician readiness for AI integration in clinical screening and diagnostic practice. The relatively large sample size and inclusion of physicians and medical interns from both governmental and private hospitals strengthen the relevance of the findings within urban hospital settings. In addition, the use of a validated questionnaire with good internal consistency strengthens the reliability of the assessment of knowledge, attitudes, and perceived barriers. The inclusion of multivariate logistic regression analysis further adds analytical rigor by allowing the identification of independent demographic factors associated with physicians’ knowledge and attitudes toward AI adoption.

Nevertheless, several limitations should be acknowledged. First, the cross-sectional design precludes the establishment of causal relationships between physicians’ characteristics and their knowledge or attitudes toward AI. Second, data were collected using a self-administered online questionnaire and may therefore be subject to response and social desirability biases, particularly given the increasing emphasis on digital transformation and AI in healthcare. Distribution of the survey through online platforms may also have favored participation by physicians who were more digitally engaged, potentially resulting in an overestimation of positive attitudes toward AI. Third, although the study included participants from multiple governmental and and private hospitals, all participants were based in Jeddah; therefore, the findings may not be fully generalizable to physicians working in other regions of Saudi Arabia, particularly rural or less digitally resourced healthcare settings. Finally, the study assessed physicians’ reported knowledge, attitudes, and perceptions rather than their actual use of AI-based tools in clinical practice.

Future research should extend these findings through longitudinal and prospective multicenter studies involving physicians from different regions of Saudi Arabia, including rural and less digitally resourced healthcare settings, to determine whether the readiness patterns observed in Jeddah are consistent across the national healthcare system. Studies should also move beyond self-reported perceptions by evaluating physicians’ actual interaction with validated AI-based screening and diagnostic tools and examining outcomes such as adoption, appropriate use, diagnostic performance, workflow efficiency, and patient safety. Given that insufficient training was the most frequently reported barrier in the present study, interventional research should evaluate the effectiveness of structured AI education, simulation-based training, workshops, and the integration of AI competencies into undergraduate, postgraduate, and continuing medical education, including whether these interventions produce sustained improvements in knowledge, confidence, trust, and appropriate clinical use. The observed association between nationality and attitudes toward AI also warrants further investigation to determine whether differences in educational background, previous exposure to digital health technologies, specialty, institutional resources, or experience with AI-enabled healthcare systems contribute to variations in acceptance. Qualitative and mixed-methods studies may further clarify the factors underlying physicians’ trust, concerns, and expectations regarding AI. Finally, future research should examine ethical, regulatory, and organizational determinants of AI implementation, including data privacy, algorithmic transparency, accountability for AI-related errors, and the appropriate balance between AI assistance and physician oversight. Addressing these areas will provide stronger evidence to guide the safe, equitable, and sustainable integration of AI into clinical screening and diagnostic practice in Saudi Arabia.

## 5. Conclusions

This study demonstrates that physicians in Jeddah, Saudi Arabia, generally hold positive attitudes toward the integration of AI in clinical screening and diagnostic practice; however, substantial gaps in knowledge and training persist. Radiology and pathology were identified as the specialties with the greatest perceived potential for AI implementation, while insufficient training and lack of trust emerged as the most significant barriers to adoption. These findings highlight physician readiness as a critical determinant of whether AI technologies can be safely and effectively translated into routine clinical care.

Addressing this readiness gap through structured education, continuous professional development, and clear regulatory and governance frameworks is essential to support responsible AI integration. Within the context of Saudi Vision 2030, strengthening physician preparedness will be key to ensuring that AI adoption enhances healthcare quality, efficiency, and patient safety in a sustainable and equitable manner.

## Acknowledgments

The authors would like to thank Lin Mazen Jolha, Shizeen Fathima Shazeer, Basila Abdul Latheef, Khadeeja Arshad sami, Layal Saeed Alghamdi, Maram Haitham AlGhazal, Rida Sharmeen Khan, Zainab Usman Ahmed, Iffat Fatima Abdullateef, Ayesha Shrouq Amin, Razan Adel Gobis, Ayesha Mirza Ayub, Abeer Osama Ismail, Husna Irfan Thalib, Rasil Fayez Alahmadi and Lujain Abdulrahim Alsharif who facilitated the dissemination of the questionnaire and gathered the data from the healthcare professionals. Also, we would like to thank all the participants who filled out the questionnaire.

## Author contributions

**Conceptualization:** Haneen Abu Alsoud, Abdulhameed Abu Alsoud, Abdulelah K. Alqawlaq, Lin Mazen Jolha, Ehab A. Abo-Ali.

**Data curation:** Abdulelah K. Alqawlaq, Abdulrahim Alissa, Malek Odah, Mohamed Abouassad, Lin Mazen Jolha, Rebal Najeeb Khayyat, Rian Saeed Yahya.

**Formal analysis:** Ehab A. Abo-Ali.

**Methodology:** Abdulelah K. Alqawlaq, Lin Mazen Jolha, Renad Ashraf Altaher, Joud Abdullah Albassam.

**Project administration:** Haneen Abu Alsoud, Abdulelah K. Alqawlaq, Ehab A. Abo-Ali.

**Software:** Ehab A. Abo-Ali.

**Supervision:** Haneen Abu Alsoud, Abdulelah K. Alqawlaq, Ehab A. Abo-Ali, Fatma E. Hassan.

**Validation:** Ehab A. Abo-Ali.

**Visualization:** Haneen Abu Alsoud, Abdulelah K. Alqawlaq, Renad Ashraf Altaher, Lujain Waleed Alqabbaa, Joud Abdullah Albassam, Ehab A. Abo-Ali, Fatma E. Hassan.

**Writing – original draft:** Haneen Abu Alsoud, Abdulhameed Abu Alsoud, Abdulelah K. Alqawlaq, Abdulrahim Alissa, Malek Odah, Mohamed Abouassad, Lin Mazen Jolha, Rebal Najeeb Khayyat, Rian Saeed Yahya, Ehab A. Abo-Ali, Fatma E. Hassan.

**Writing – review & editing:** Haneen Abu Alsoud, Abdulhameed Abu Alsoud, Abdulelah K. Alqawlaq, Renad Ashraf Altaher, Lujain Waleed Alqabbaa, Joud Abdullah Albassam, Fatma E. Hassan.


