# Supplementary material for: Physician readiness for artificial intelligence integration in clinical screening and diagnosis: Multicenter cross-sectional study in Jeddah, Saudi Arabia
Source: Medicine (Baltimore). 2026 Sep 25;105(39):e50871. doi: 10.1097/MD.0000000000050871 (PMC13619217; doi:10.1097/MD.0000000000050871)
Supplement: Supplementary file 1 [file medi-105-e50871-s001.docx]

**Physician Readiness for Artificial Intelligence Integration in Clinical Screening and Diagnosis: A Multicenter Study in Jeddah, Saudi Arabia**

We are a group of medical students conducting research to evaluate **the Awareness and Perceptions of AI in Medical Screening and Diagnosis Among Healthcare Professionals a cross-sectional study in Jeddah Saudi Arabia**. If you read this form and agreed to participate, please understand your participation is voluntary and anonymous, your privacy will be maintained in all published and written data resulting from the study. Participation may not benefit you directly, but it will help us for the research purposes.

If you have any questions regarding this survey or wish to obtain the protocol for this research project, please contact:

Dr. Haneen Abualsoud: [Haneenluay@hotmail.com](mailto:Haneenluay@hotmail.com)

**Applied questionnaire:**

Section A: Consent

1. By agreeing to participate in this study you are a practicing physician.
   - Yes
   - No

Section B: Demographics

1. Do you live in Jeddah?
   - Yes
   - No
2. Nationality
   - Saudi
   - Non-Saudi
3. Gender?
   - Male
   - Female
4. Age: (In Years)
   - 25-34
   - 35-44
   - 45- and more
5. What is your job title?

- Intern
- General Practitioner (GP)
- Specialist
- Residence
- Consultant

6. How many years of experience do you have in the medical field?
   - Below 5 years
   - 5-10 years
   - Above 10 years

Section C: Knowledge

7. Did you hear about AI before?
   - Yes
   - No
8. If yes, from where did you hear?
   - Workshop
   - Colleague
   - Event
   - Conference
   - Online course
   - Internet
   - Other
9. Artificial intelligence (AI) is used in medical diagnosis and screening.
   - True
   - False
   - Not sure
10. AI can be used to analyze medical images such as X-rays, MRIs, or CT scans for diagnosing conditions.
    - True
    - False
    - Not sure
11. AI has been shown to assist healthcare professionals in diagnosing medical conditions more accurately than traditional methods.
    - True
    - False
    - Not sure
12. AI systems have been demonstrated to identify diseases or conditions that are difficult for human doctors to detect.
    - True
    - False
    - Not sure
13. AI is currently capable of independently diagnosing medical conditions without human oversight, according to existing medical practices and research?
    - True
    - False
    - Not sure

Section D: Attitude

14. I believe AI has the potential to significantly improve diagnostic accuracy in medical practice.
    - Strongly agree
    - Agree
    - Neutral
    - Disagree
    - Strongly disagree
15. I am confident in the ability of AI-based systems to assist in diagnosing medical conditions within my specialty.
    - Strongly agree
    - Agree
    - Neutral
    - Disagree
    - Strongly disagree
16. I believe AI has the ability to reduce the workload for healthcare professionals.
    - Strongly agree
    - Agree
    - Neutral
    - Disagree
    - Strongly disagree
17. I believe AI can enhance the efficiency of patient care
    - Strongly agree
    - Agree
    - Neutral
    - Disagree
    - Strongly disagree
18. I believe AI should be used only as an assistive tool, with healthcare professionals making the final decisions
    - Strongly agree
    - Agree
    - Neutral
    - Disagree
    - Strongly disagree
19. I believe AI should replace certain tasks currently performed by human doctors
    - Strongly agree
    - Agree
    - Neutral
    - Disagree
    - Strongly disagree
20. I believe AI-based tools should be used in clinical practice for diagnosing or screening patients
    - Strongly agree
    - Agree
    - Neutral
    - Disagree
    - Strongly disagree
21. Is it ethical important to inform patients about the use of AI in their diagnostic process?
    - Strongly agree
    - Agree
    - Neutral
    - Disagree
    - Strongly disagree
22. Is it ethical to determine who is responsible if AI-based diagnostic tools make an incorrect diagnosis?
    - Strongly agree
    - Agree
    - Neutral
    - Disagree
    - Strongly disagree

Section E: Facilitators and barriers and future potential

23. Which of the following diagnostic areas do you think AI has the most potential in?
    - Radiology
    - Pathology
    - Oncology
    - Cardiology
    - Neurology
    - Primary care
    - Mental health
    - Surgery
24. What do you think are the biggest barriers to AI adoption in your clinical practice? (Select all that apply)
    - Cost of AI tools
    - Lack of trust in AI systems
    - In sufficient training or education
    - Legal or regulatory barriers
    - Concerns About data security and patient privacy
    - Lack of clear evidence of AI efficacy
    - Resistance from colleagues or leadership
25. What do you think are the biggest facilitators to AI adoption in your clinical practice? (Select all that apply)

- Improved patient outcomes
- Enhanced efficiency and workflow optimization
- Access to comprehensive data analytics
- Support from leadership and organizational culture
- Regulatory frameworks and standards
- Interdisciplinary collaboration
- Training and education

26. What is the best way to familiarize health care professionals to use AI tools?
    - Workshops / Training Sessions
    - Online Courses / Webinars
    - AI-Focused Conferences and Seminars
    - Research and Publications
    - Integration of AI into Medical School or Residency Curricula
    - Rewards and Recommendations for Adoption and Use of AI Tools
    - Incorporating Healthcare Professionals’ Feedback and Iterative Improvements on AI Tools
    - Collaborations with AI Developers or Suppliers
27. Who do you believe should be responsible if AI-based diagnostic tools make an incorrect diagnosis?

- The healthcare provider
- The AI developer
- The institution or hospital
- No one
